# Inhibition of Progenitor Dendritic Cell Maturation by Plasma from Patients with Peripartum Cardiomyopathy: Role in Pregnancy-associated Heart Disease

**DOI:** 10.1080/17402520500304352

**Published:** 2005-12

**Authors:** Jane E. Ellis, Aftab A. Ansari, James D. Fett, Robert D. Carraway, Hugh W. Randall, Mario I. Mosunjac, J. Bruce Sundstrom

**Affiliations:** 1 Department of Gynecology and Obstetrics Emory University School of Medicine Atlanta GA 30322 USA; 2 Department of Pathology and Laboratory Medicine Department of Pathology and Laboratory Medicine Atlanta GA 30322 USA; 3 Department of Adult Medicine Deschapelles Hospital Albert Schweitzer Haiti

## Abstract

Dendritic cells (DCs) play dual roles in innate and adaptive immunity based on their functional maturity, and both innate and adaptive immune responses have been implicated in myocardial tissue remodeling associated with cardiomyopathies. Peripartum cardiomyopathy (PPCM) is a rare disorder which affects women within one month antepartum to five months postpartum. A high occurrence of PPCM in central Haiti (1 in 300 live births) provided the unique opportunity to study the relationship of immune activation and DC maturation to the etiology of this disorder. Plasma samples from two groups (*n* = 12) of age- and parity-matched Haitian women with or without evidence of PPCM were tested for levels of biomarkers of cardiac tissue remodeling and immune activation. Significantly elevated levels of GM-CSF, endothelin-1, proBNP and CRP and decreased levels of TGF- were measured in PPCM subjects relative to controls. Yet despite these findings, in vitro maturation of normal human cord blood derived progenitor dendritic cells (CBDCs) was significantly reduced (*p* < 0.001) in the presence of plasma from PPCM patients relative to plasma from post-partum control subjects as determined by expression of CD80, CD86, CD83, CCR7, MHC class II and the ability of these matured CBDCs to induce allo-responses in PBMCs. These results represent the first findings linking inhibition of DC maturation to the dysregulation of normal physiologic cardiac tissue remodeling during pregnancy and the pathogenesis of PPCM.


*Clinical & Developmental Immunology*, December 2005; 12(4): 265–273

Taylor & Francis

Taylor & Francis Group

### Inhibition of progenitor dendritic cell maturation by plasma from patients with peripartum cardiomyopathy: Role in pregnancy-associated heart disease*

JANE E. ELLIS[1], AFTAB A. ANSARI[2], JAMES D. FETT[3], ROBERT D. CARRAWAY[3], HUGH W. RANDALL[1], MARIO I. MOSUNJAC[2], & J. BRUCE SUNDSTROM[2]

[1]*Department of Gynecology and Obstetrics, Emory University School of Medicine, Atlanta, GA 30322, USA,* [2]*Department of Pathology and Laboratory Medicine, Emory University School of Medicine, Atlanta, GA 30322, USA, and* [3]*Department of Adult Medicine, Hospital Albert Schweitzer, Deschapelles, Haiti*

**Abstract**
Dendritic cells (DCs) play dual roles in innate and adaptive immunity based on their functional maturity, and both innate and adaptive immune responses have been implicated in myocardial tissue remodeling associated with cardiomyopathies. Peripartum cardiomyopathy (PPCM) is a rare disorder which affects women within one month antepartum to five months postpartum. A high occurrence of PPCM in central Haiti (1 in 300 live births) provided the unique opportunity to study the relationship of immune activation and DC maturation to the etiology of this disorder. Plasma samples from two groups ($n = 12$) of age- and parity-matched Haitian women with or without evidence of PPCM were tested for levels of biomarkers of cardiac tissue remodeling and immune activation. Significantly elevated levels of GM-CSF, endothelin-1, proBNP and CRP and decreased levels of TGF-β were measured in PPCM subjects relative to controls. Yet despite these findings, *in vitro* maturation of normal human cord blood derived progenitor dendritic cells (CBDCs) was significantly reduced ($p < 0.001$) in the presence of plasma from PPCM patients relative to plasma from post-partum control subjects as determined by expression of CD80, CD86, CD83, CCR7, MHC class II and the ability of these matured CBDCs to induce allo-responses in PBMCs. These results represent the first findings linking inhibition of DC maturation to the dysregulation of normal physiologic cardiac tissue remodeling during pregnancy and the pathogenesis of PPCM.

**Keywords:** *Cardiomyopathy, dendritic cells, innate immunity, pregnancy*

### Introduction

Peripartum cardiomyopathy (PPCM) is a rare disease in the United States with a reported incidence between 1:3 000 and 1:15 000 pregnancies (Ventura et al. 1997, Brown and Bertolet 1998). The four primary diagnostic criteria are (a) development of cardiac failure in the last month of pregnancy or within five months postpartum; (b) absence of an identifiable cause for the cardiac failure; (c) absence of recognizable heart disease prior to the last month of pregnancy and (d) left ventricular dilatation and decreased contractility demonstrated by echo criteria of depressed fractional shortening (<30%) or ejection fraction (<45%). Approximately 20% of women with PPCM die unless they receive cardiac transplant. During normal pregnancy increased hemodynamic demand and environmental stress associated with late stage pregnancy initiate physiological compensatory responses that involve well-regulated homeostatic structural remodeling of decidual and cardiovascular tissues. Cellular components of the innate arm of the immune system mediate such remodeling responses by assisting in the clearance and resorption of dead or dying cells and by helping to create supporting

Correspondence: J. Bruce Sundstrom, Department of Pathology and Laboratory Medicine, Emory University School of Medicine, WMB Room 2335, 101 Woodruff Circle, Atlanta, GA 30322, USA. Tel: 1 404 712 2837. Fax: 1 404 712 1771. E-mail: jsundst@emory.edu
*This work was supported in part by Emory University Research Committee award # 2002081.

ISSN 1740-2522 print/ISSN 1740-2530 online © 2005 Taylor & Francis
DOI: 10.1080/17402520500304352

networks of connective tissue and vascular beds. Under physiological conditions these cellular responses are regulated in part by the coordinated local release of specific chemokines, cytokines and growth factors that form a gradient that leads to the recruitment and development of bone marrow derived progenitors which mediate tissue remodeling and repair in the tissues secreting the mediators without triggering unwanted immunopathology. Although, the exact etiology of PPCM remains unknown, infectious, inflammatory, genetic, hormonal and/or metabolic co-factors may contribute to a disruption of these physiologically regulated pregnancy-associated cardiac tissue remodeling responses which if not reversed leads to cardiomyopathy. It is to be noted that such abnormalities are indeed reversible since a significant number of PPCM patients do return to normal cardiac function. Thus, the reasons for these divergent clinical outcomes identify one of the major issues that needs to be determined.

Thus, we submit that the lack of a physiologically normal remodeling response facilitates myocardial tissue injury which can result in the release of certain cryptic cardiac antigens, e.g. myosin, mitochrondrial proteins, natriuretic peptides, heat shock proteins (HSPs), etc. that can become exposed to immature dendritic cells (DCs), which under the influence of other "danger signals" present during the injury and remodeling process (such as CD40L expressed on activated platelets (Stumpf et al. 2003) undergo maturation and become able to initiate subsequent cardiac-specific auto-immune responses which may contribute significantly to further cardiac tissue injury. Cardiac tissue remodeling can also be mediated by a variety of endogenous stress induced proteins including, TNF-$\alpha$, IL-1, IL-6, endothelin-1, angiotensin II, matrix metalloproteinases (Kai et al. 1998, Schwartzkopff et al. 2002, Altieri et al. 2003, Inoue et al. 2003, Yamazaki et al. 2004), TGF-$\beta$, acidic and basic fibroblast growth factors, vascular endothelial growth factor (VEGF) and others (Mann 2002a, b, 2003, Zolk et al. 2002, Flesch et al. 2003) released locally by cardiovascular tissues. These stress induced proteins act directly on both cardiac myocytes to induce hypertrophy, cytoprotection or repair, and on non-myocyte cardiac tissues to increase vascularization and to effect ECM remodeling and fibrosis.

The recent identification of a significantly high incidence and prevalence of PPCM among women in the Hospital Albert Schweitzer (HAS) District in central Haiti (Fett et al. 2002) presented an unusual opportunity to study the potential mechanism(s) of this disease. The objectives of the present study were to (1) assess the role of innate immunity in the pathogenesis of PPCM by characterizing differences in the levels of stress-induced proteins associated with innate immune-mediated tissue remodeling measured in plasma isolated from Haitian PPCM patients relative to age- and parity-matched Haitian mothers without disease and (2) to measure the functional potential of plasma from PPCM and control Haitian subjects to induce the maturation of cord blood derived progenitor dendritic cells (CBCDs) which we reason is involved in the remodeling response. The results presented herein indicate that despite significant elevation in plasma levels of biomarkers of cardiac tissue remodeling and immune activation, plasma from Haitian PPCM patients exhibit a significantly reduced potential for inducing DC maturation, an event required for physiological tissue remodeling, and thus for the first time provide evidence for a pathogenic role for DC function and dysregulation of innate immunity in the etiology of PPCM in this patient group.

### Methods

### *Patients and clinical specimens*

The subjects enrolled in the study were Haitian mothers from the HAS District in Dechappelles, Haiti, where a high incidence of PPCM has been reported and a PPCM registry has recently been established (Fett et al. 2002). The criteria for patient enrollment in the HAS PPCM Registry are based on the following case definition: (1) onset of congestive heart failure during the period of one month before delivery through five months postpartum; (2) absence of preexisting heart disease; (3) no other identifiable cause of congestive heart failure—this involves ruling out other causes of CHF, including but not limited to severe anemia, sepsis, severe toxemia of pregnancy, tuberculosis or other pulmonary diseases, HIV-1, beriberi heart disease or other nutritional deficiencies, tocolytic agents or other toxic exposures, fluid overload, amniotic fluid embolism, valvular heart disease, coronary artery disease, hypertensive cardiovascular disease, thyrotoxicosis or other endocrinopathies and familial dilated cardiomyopathy and (4) evidence of left ventricular dilatation with diminished contractility characterized by an ejection fraction less than 45% or fractional shortening less than 30% and end-diastolic dimension more than 2.7 $cm/M^2$ as determined by echocardiography. In addition, the following documentation was included for each patient in the registry: history and physical exam, New York Heart Association functional classification (I–IV), chest X-ray, echocardiogram, HIV serology (positive serologies were excluded from the registry), RPR as part of prenatal care, urinalysis, hematocrit and an epidemiology questionnaire. Parity-matched non-PPCM Haitian mothers from the same (HAS) hospital district were selected from the Women's Health Registry and invited to participate in the study as controls. Following informed consent, a 20 ml sample of whole blood was collected in vacutainer collection tubes containing EDTA as an anti-coagulant. After centrifugation, the plasma was aliquoted and stored at − 20°C.

Human umbilical cord blood was collected following delivery of term fetuses of mothers with no known health or pregnancy related complications who delivered at Grady Memorial Hospital in Atlanta, GA. Cord blood was collected in sterile 50 ml centrifuge tubes containing 5 ml of sterile Hank's buffered saline solution supplemented with 500 units of sodium heparin and 2.5 mg of gentamicin. The blood was gently mixed and stored for less than 24 h at room temperature. Mononuclear cells were isolated from umbilical cord blood by density gradient centrifugation on lymphocyte separation medium. CD34 + cord blood stem cells (CBSCs) were then positively separated in a magnetic field using EasySep immunomagnetic beads (Stem Cell Technologies, Vancouver, BC), The collection of all clinical specimens was conducted under established protocols approved by IRBs at HAS and Emory University.

### *Determination of serum levels of biomarkers for heart disease and innate immune activation*

Plasma levels for a select group of biomarkers associated with left ventricular remodeling, DC maturation and innate immune activation, including proBNP, C-reactive protein, endothelin-1, antibodies to HSP 60 and 70, IL-1α, IL-1β, IL-4, IL-6, IL-10, CD40L, GM-CSF, TNF-α and TGF-β, were measured using commercially available immunoassays (Stressgen Biotechnologies Corp., Victoria BC, Canada; Biosource International, Camarillo California; and APLCO, Windham, NH) according to their manufacturer's instructions.

### *Proliferation assays*

Mature DCs, unlike immature progenitor DCs, richly express MHC class II and select costimulatory molecules, e.g. CD80 and 86, and based on the spectrum of peptides bound to their MHC II molecules are able to engage in contact dependent interactions with cognate T cells resulting in readily measurable T cell proliferation. Thus, *in vitro* co-culture assays were used to assess the ability of plasma from PPCM or control subjects to induce the functional maturation of cord blood derived progenitor immature DCs (prCBDCs) and following their maturation assess their ability to induce (allo-) proliferation of HLA miss-matched PBMC responders. Pooled CBDCs were developed from CD34 + stem cells (CBSCs) isolated from human cord blood as described and then suspended at $10^6$/ml in serum free stem cell medium supplemented with 2 mM L-glutamine, and gentamicin (50 μg/ml). To promote the development of prCBDCs, stem cell medium was supplemented with 2 ng/ml GM-CSF; to promote the development of mature CBDCs (positive controls), stem cell medium was supplemented with GM-CSF (10 ng/ml), LPS (1 μg/ml) IL-4 (10 ng/ml), and TNF-α (2 000 U/ml); and to evaluate the effect of PPCM or control plasma on prCBMC maturation, CBSCs were cultured in stem cell medium supplemented with 2 ng/ml GM-CSF and 25% (v/v) human plasma isolated from (a) pooled fetal cord blood, (b) normal non-Haitian—nulliparous adults, (c) normal age and parity matched Haitian adult females or (d) Haitian PPCM donors. For each culture condition CBSCs were incubated in a humidified environment at 37°C with 7% $CO_2$ for 4–7 days. CBDCs were then harvested, counted and re-suspended at $10^5$/ml in RPMI media supplemented with 10% FBS without recombinant growth factors. A total of $10^4$ prCBDCs was then placed in co-culture with $10^5$ ficol-hypaque—purified allogeneic PBMCs from normal donors in a total volume of 0.2 ml of media in triplicate wells in round-bottomed 96-well tissue culture plates then incubated as described for an additional 96 h. Each well was then pulsed with 1 μCi/well of methyl-[$^3$H]- thymidine (2Ci/mmol, NEN, Boston, MA) for 16 h before harvesting and counting for 1 min on a Wallac MicroBeta TriLux liquid scintillation counter (PerkinElmer Life Sciences, Boston MA). Controls consisted of prCBDCs, CBDCs or PBMCs cultured alone in media. Quantitative measurements of cell proliferation (mean counts per minute [cpm]) were normalized by subtracting mean cpm from cultures of CBDCs without PBMCs.

### *Flow microfluorometry (fmf) analysis*

Detection of cell surface-expressed antigens associated with a phenotype of either immature or mature DCs was performed by FMF using fluorochrome conjugated mouse anti-human monoclonal antibodies to HLA-DR, CD80, CD83, CD86 and CD11a (CCR7). Aliquots of progenitor DCs were incubated in individual 12 × 75 mm plastic tubes with 1 μg of Phycoerythrin (PE) conjugated HLA-DR (MHC Class II), MHC Class I, CD80, CD83, CD86 or CCR7 (Becton Dickinson Immunocytometry Systems, Pleasantville, CA) in media for 30 min on ice then washed twice with Dulbecco's PBS pH 7.4 and immediately analyzed by FMF with a Becton Dickinson FACS-Caliber using cellquest imaging software.

### *Statistical analysis*

Statistical analysis of replicate measurements of independent samples taken at a single or multiple time points was performed after descriptive tests (e.g. the Shapiro–Wilk W test) were conducted to verify that the observed measurements were normally distributed. Differences between means of multiple independent normally distributed samples were assessed by one way analysis of variance (ANOVA) with contrasts and $p$ values were assigned using Tukey's 95% confidence intervals. Non-parametric tests (Mann–Whitney rank sum tests) were employed on all measurements where a normal distribution of the observation was not assumed. All statistical analyses was performed using Analyze-It software V. 1.62 for Microsoft Excel (Leeds, UK).

### Results

*Baseline clinical profiles of haitian study subjects*

The baseline clinical data for the PPCM ($n = 12$) and control ($n = 12$) patients participating in this study are presented in Table I. The range and median values for age, time post-partum of plasma collection, gravida, parity, left ventricular ejection fraction (LVEF), LV end-diastolic diameter (LVEDD), LV shortening fraction (LVSF), LV end-diastolic diameter/meter-squared body surface area (EDD/$M^2$), and New York Heart Association (NYHA) functional classification are presented for both study subject groups. Non-parametrical analysis revealed that the two study groups did not differ significantly in age, time of specimen collection post-partum, gravida, or parity. However, as expected the PPCM subjects did differ significantly ($p < 0.04$) from control subjects in their values for LVEF and LVEDD and in their NYHA functional classification.

*Disease-specific differences in plasma levels of biomarkers of inflammation and cardiac tissue remodeling*

Brain Natriuretic Peptide (BNP) belongs to a family of hormones which includes Atrial Natriuretic Peptide (ANP), and C-type Natriuretic Peptide (CNP), all of which are secreted by the atrium, ventricle and cardiac microvascular endothelial cells(Suzuki et al. 2001). BNP is derived from its high MW precursor, pro-BNP (1–108 AA) and functions to regulate vascular tone through a spectrum of hypotensive effects which protect against fluid overload and high blood pressure. The mature form, BNP-32, consists of the 32 C-terminal AA residues (77–108) of the parent pro-BNP, both of which are stored in ventricular tissues. It has been suggested that elevated plasma levels of amino-terminal pro-BNP (Nt-pro-BNP) is a better indicator of early cardiac dysfunction than BNP-32. Plasma levels of pro-BNP for 12 PPCM patients and 12 control subjects used in this study are displayed in Table II. Statistically significant differences between the two groups ($p < 0.0001$) was determined by both one-way ANOVA and by independent samples $t$-test as described in the methods section.

C-Reactive protein is an acute phase pentameric globulin produced by the liver in response to inflammation and tissue injury. Plasma levels have been reported to be significantly elevated in patients with advanced DCM or lymphocytic myocarditis and to correlate with acute left ventricular decompensation (Kaneko et al. 1999, 2002). Measurement of plasma CRP values has also been shown to be a predictor of improvement or readmission during heart failure (Alonso-Martinez et al. 2002). Plasma levels of CRP in Haitian PPCM patients ($n = 12$) relative to control subjects ($n = 12$), measured by capture ELISA as described in the methods section, were found to be significantly elevated ($p < 0.002$) by ANOVA (Table II).

Stress due to hyperthermia or hypoxia can result in the rapid denaturing of cellular proteins including mitochrondrial proteins vital to cellular metabolism and survival. HSPs are specialized proteins that possess an intrinsic chaperone function and can

Table I. Baseline clinical data on PPCM and control subjects.

| | PPCM | | | Control | | |
|---|---|---|---|---|---|---|
| | Median | Range | $n$ | Median | Range | $n$ |
| Age | 25 | 17–40 | 12 | 28.5 | 16–38 | 12 |
| Time post-partum (days) | 49 | 19–101 | 12 | 128 | 65–194 | 12 |
| Gravida | 3.2 | 1–7 | 12 | 3 | 1–10 | 12 |
| Parity | 3 | 1–7 | 12 | 3 | 1–10 | 12 |
| LVEF (%)*,† | 25 | 15–30 | 12 | 50 | 40–55 | 12 |
| LVEDD†,‡ | 6.15 | 5.2–7.2 | 12 | 4.1 | 4–5.6 | 12 |
| LVSF¶ | 15 | 5–24 | 12 | 30 | 20–35 | 12 |
| EDD/M§ | 4.05 | 3.5–4.8 | 12 | 2.7 | 2.7–3.7 | 12 |
| NYHA functional class‖ | III | III–1V | 12 | I | I | 12 |

* LVEF = left ventricular ejection fraction meets ppcm criteria if less than 45%, normal = 50% and >.
† Values for PPCM significantly different from control ($p < 0.04$).
‡ LVEDD = left ventricular end-diastolic diameter.
¶ LVSF = left ventricular shortening fraction meets ppcm criteria if less than 30%, normal = 35% and >.
§ EDD/$M^2$ = left ventricular end-diastolic diameter/meter-squared body surface area, normal 2.7 cm/$M^2$ or less.
‖ NYHA = New York Heart Association functional classification.

Table II. Biomarkers of inflammation and remodeling.

| | Endothelin I (pg/ml) | | proBNP (fmol/ml) | | CRP (Mg/ml) | | Anti-HSP60* (ng/ml) | | Anti-HSP70* (ng/ml) | |
|---|---|---|---|---|---|---|---|---|---|---|
| Group | Control | PPCM | Control | PPCM | Control | PPCM | Control | PPCM | Control | PPCM |
| Mean | 0.75 | 1.823 | 74.77 | 413.8 | 3.97 | 201.2 | 37.419 | 27.161 | 3.595 | 2.354 |
| *t*-statistic | 7.5 | | 3.44 | | 3.47 | | | | | |
| Two-tailed *t* | < 0.0001 | | < 0.0054 | | < 0.0052 | | ND | | ND | |

*values represent plasma levels (ng/ml) of total antigen-specific IgG, IgA and IgM.

facilitate the proper folding (or refolding) of (denatured) proteins. The rapid expression of HSPs in response to physiologic stress thus helps to protect cells from injury (Hendrick and Hartl 1995, Bukau and Horwich 1998). Interaction between HSP and certain pattern recognition receptors (PRR) that recognize conserved epitopes termed pathogen-associated molecular patterns causes non-specific activation and maturation of PRR- expressing progenitor DCs (Kuppner et al. 2001). Through such interactions HSPs are able to activate the innate immune system. It has been proposed that such events ultimately result in adaptive immune responses manifested by the appearance of antibodies directed against HSP and HSP associated cellular proteins (Latif et al. 1993, Portig et al. 1997, Srivastava 2002a, b). Elevated serum levels of certain HSPs, e.g. HSP60 and HSP70, as well as anti-HSP antibodies are associated with cardiovascular diseases including DCM (Latif et al. 1993, 1999, Knowlton et al. 1998, Mandi et al. 2000). The levels of antibodies to HSP60 and HSP-70 were measured in plasma samples from both PPCM and Control subjects as described in the methods section. However, no statistically significant differences were detected (Table II).

*Disease-specific differences in plasma levels of biomarkers of innate immune activation*

Several stress-activated cytokines have been shown to play important biochemical roles in initiating responses to cardiac tissue injury induced by increased hemodynamic demands to preserve and maintain heart function during environmental or physiological stress (Mann 1996, Aukrust et al. 1999, Anker and von Haehling 2004). Such cytokines are known to have pleiotropic effects and may positively influence (IL-1β, TNF-α, GM-CSF, IL-4, IFN-γ) or negatively influence (TGF-β and IL-10) maturation of progenitor DCs. Prolonged activation or over-expression of such cytokines may lead to pathological decompensation. Plasma levels of a select group of these cytokines were measured by capture ELISA as described in the methods section and are shown in Table III. No disease specific differences were observed in plasma levels of IL-1α, IL-4, IFN-γ or IL-10. While elevated levels of IL-1β ($p < 0.06$), TNF-α ($p < 0.08$) and sCD40L

Table III. Plasma levels of cytokines and growth factors involved in DC maturation.

| Cytokine | IL-1β* (pg/m/) | | TNF-α* | | SCD40L* | | GM-CSF* | | TGF-β† | |
|---|---|---|---|---|---|---|---|---|---|---|
| Group | Control | PPCM | Control | PPCM | Control | PPCM | Control | PPCM | Control | PPCM |
| | | | | | *Disease-specific differences* | | | | | |
| *n* | 12 | 12 | 12 | 12 | 12 | 12 | 12 | 12 | 12 | 12 |
| Mean | 0.00 | 2.75 | 1.27 | 2.03 | 0.00 | 2.13 | 0.00 | 2.74 | 0.76 | 0.58 |
| *t*-Statistic | − 2.01 | | − 1.82 | | − 1.49 | | − 2.97 | | 2.09 | |
| DF | 22.0 | 22 | 22.0 | 22 | 22.0 | 22 | 20.0 | | 22.0 | |
| Two-tailed *t* | 0.06 | | 0.08 | | 0.15 | | 0.0076‡ | | 0.05‡ | |
| Cytokine | IL-1α (pg/m/)* | | IL-4 (pg/m/)* | | IFN-γ* (pg/m/) | | IL-10† | | | |
| Group | Control | PPCM | Control | PPCM | Control | PPCM | Control | PPCM | | |
| | | | | | *No-disease-specific differences* | | | | | |
| *n* | 12 | 12 | 12 | 12 | 12 | 12 | 12 | 12 | | |
| MEAN | | | | | | | | | | |
| *t*-statistic | | | | | | | | | | |
| DF | 22 | 22 | 22 | 22 | 22 | 22 | 22 | 22 | | |
| Two-tailed *t* | ND | | ND | | ND | | ND | | | |

* Factors that promote DC maturation.
† Factors that suppress DC maturation.
‡ Statistically significant differences.

($p < 0.15$) were measured in PPCM plasma these values did not achieve statistical significance. The only 2 cytokines that showed significant differences were elevated plasma levels of GM-CSF ($p < 0.0076$) and diminished levels of TGF-β ($p < 0.05$) in PPCM subjects relative to control subjects (Table III).

*Allo-proliferative responses to cord blood derived dendritic cells*

It has been previously shown that chemokines attract progenitor cells of the dendritic cell lineage (pDCs) to sites of tissue injury and in the presence of select cytokines and growth factors such pDCs develop into mature DCs. Mature DCs richly express MHC class II and the co-stimulatory molecules, such as CD80 and CD86 which supports their functional ability to present cognate antigens and induce proliferative responses in antigen specific T cells and proliferative responses in allogeneic T cells in standard mixed lymphocyte cultures. It was reasoned that if PPCM patients were experiencing tissue injury such injury could lead to the synthesis and release of cytokines and growth factors in their plasma that would facilitate the trafficking, migration and maturation of pDCs into fully competent functionally mature DCs. In order to test this hypothesis, allo-proliferative responses of PBMCs to cord blood derived prDCs cultured in the presence of plasma from PPCM patients and for purposes of control plasma from age and parity matched Haitian women without evidence of heart disease were measured in MLRs to assess the functional maturation of CBDCs. To first establish the optimal kinetics and culture conditions for full functional maturation of progenitor CBDCs in vitro, purified cord blood derived CD34 + cells were incubated in serum-free stem cell media supplemented with low dose (2 ng/ml) GM-CSF alone to promote the development of immature progenitor DC or with high dose GM-CSF (10 ng/ml) plus IL-4 (10 ng/ml), TNF-α (10 ng/ml) and LPS (1 μg/ml) to promote the development of mature DCs. After 4 and 7 days in culture, the cord blood cells were resuspended in RPMI-10 media with 10% fetal bovine serum (FBS) and then serially diluted in 100 μl volumes in replicate wells in a microtiter tissue culture plate to serve as stimulator cells in a 96-H allo-proliferation assay as described in the methods section. A total of $10^5$ allogeneic PBMCs obtained from two healthy donors were added to each well to serve as responder cells. The results from these proliferation experiments demonstrate that by 4 or 7 days cord blood derived CD34 + cells cultured in media supplemented with low dose (2 ng/ml) GM-CSF alone were unable to induce significant proliferative responses in PBMCs. However, by 7 day, as few as 1000 cord blood derived CD34 + cells cultured in media supplemented with GM-CSF (10 ng/ml) plus IL-4 (10 ng/ml), TNF-α (10 ng/ml), and LPS (1 μg/ml) were able to induce significant allo-proliferative responses in PBMCs (Figure 1A). Thus, it was reasoned that if plasma from mothers post partum would normally contain such growth factors that would promote functional maturation of progenitor DCs required for normal physiologic remodeling peripartum, the question remained whether such growth factors would also be present in plasma from PPCM patients.

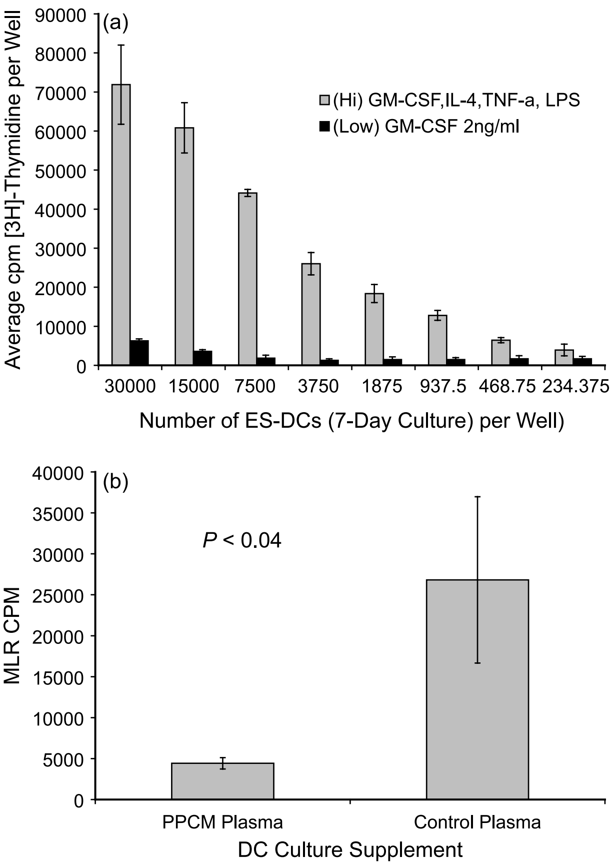

Figure 1. (a) Human cord blood derived dendritic cells induce allo-proliferation of PBMCs. Cord blood derived CD34 + cells were cultured in serum-free stem cell media supplemented with low dose (2 ng/ml) GM-CSF (Low) alone to promote development of immature progenitor DC or with high dose GM-CSF (10 ng/ml) plus IL-4 (10 ng/ml), TNF-α (10 ng/ml) and LPS (1 μg/ml) (Hi) to promote development of mature DC. Cell (allo-) proliferation assays (MLR) were conducted for 96 h with two sets of MHC disparate PBMC responders. Allo-proliferative responses to 7 day cultured cord blood progenitor DCs are represented as the mean cpm of incorporated [3H]-thymidine from triplicate cultures. (b) PPCM plasma does not promote DC maturation. Cord blood derived CD34 + cells were cultured for 7 days in serum-free stem cell media supplemented with low dose (2 ng/ml) GM-CSF and 25% (v/v) plasma from control patients ($n = 7$) or PPCM patients ($n = 7$) to induce differentiation and maturation of DCs. Afterwards, *in vitro* differentiated DCs were cocultured with PBMCs isolated from normal donors and allo-proliferative responses were determined by measuring cpm of incorporated [$H^3$]-thymidine as described.

*Allo-proliferative responses of PBMCS to human cord blood- derived dendritic cells developed in PPCM vs control plasma*

Proliferation experiments were thus subsequently conducted with progenitor CBDCs and HLA mismatched PBMCs as described in the methods section. Proliferative responses of PBMCs co-cultured with HLA mismatched progenitor CBDCs in media supplemented with low dose (2 ng/ml) GM-CSF and 25% v/v plasma from normal non-Haitian—nulliparous volunteers or pooled cord blood were below detectable limits (data not shown). However, 7 out of 12 PPCM plasma samples and 7 out of 12 Haitian control plasma samples did induce DC maturation as reasoned above leading to detectable allo-proliferative responses in PBMCs. However, of importance was the finding that the magnitude of allo-proliferative responses to DCs cultured in Haitian control plasma was significantly greater ($p < 0.04$) than proliferative responses to CBDCs cultured in PPCM conditioned media (Figure 1B).

*Phenotypic characterization of CBDC maturation by FMF*

It was reasoned that the lower levels of allo-proliferative responses to prCBDCs developed in PPCM plasma was either due to lack of appropriate frequency and density of MHC/co-stimulatory molecule expression or perhaps due to intrinsic properties of the CBDCs developed in the presence of plasma isolated from PPCM patients. In efforts to address this issue, progenitor CBDCs were cultured in triplicate in media supplemented with low dose (2 ng/ml) GM-CSF and 25% v/v plasma from PPCM or control subjects ($n = 12$). After 7 days, the cells were harvested and the levels of cell surface expressed molecules associated with mature DCs were measured by FMF as described in the method section. The levels of expression of MHC class I and II, CD80 and 86 costimulatory molecules, and CD83 and CCR7 DC maturation antigens were all significantly lower ($p < 0.0001$) on CBDCs cultured in the presence of PPCM plasma relative to controls (Figure 2). These data provide further evidence that plasma from PPCM patients as compared to plasma from control subjects does not support prDC maturation.

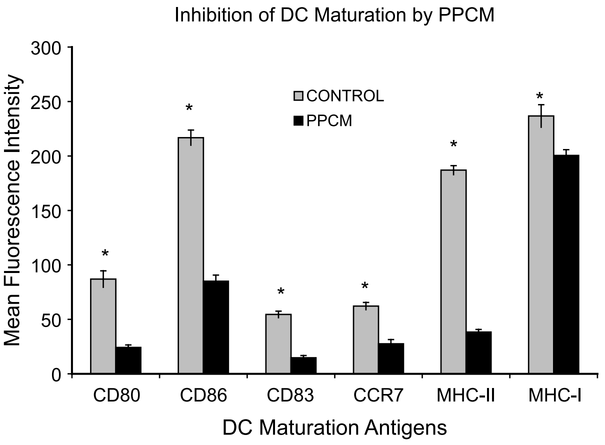

Figure 2. Analysis of DC maturation markers FMF. Cord blood derived CD34 + cells were cultured for seven days in serum-free stem cell media supplemented with low dose (2 ng/ml) GM-CSF and 25% (v/v) plasma from control patients ($n = 7$) or PPCM patients ($n = 7$) to induce differentiation and maturation of DCs. *In vitro* differentiated DCs were then subjected to FMF to determine the relative expression antigens associated with DC maturation: CD80, CD86, CD83, CCR7, MHC class II and MHC class I. Levels of expression are represented as mean channel fluorescence intensity.

### Discussion

Cells of the innate arm of the immune system, such as DCs and mast cells arise from bone-marrow derived hematopoietic precursors. Their ontological development and activation is determined and regulated by environmental signals generated in part in response to a variety of infectious and non-infectious agents. Our hypothesis is that during normal pregnancy, remodeling of the heart is required for return to normal physiological status. This remodeling requires cells of different lineages including phagocytic DCs whose progenitors are attracted to the cardiac tissue by the synthesis and release of a gradient of chemokine and growth factors that induce their functional maturation. Thus, plasma from such peri/postpartum normal pregnant mothers contain a spectrum of such chemokines, cytokines and growth factors able to mediate the recruitment and development of different populations of undifferentiated and specialized cells of hematopoietic origin. Initially, appropriate chemokines for the recruitment and development of cells responsible for the removal of dead or dying cells and debris are expressed followed by factors necessary for the recruitment and development of progenitor cells that possess the ability to participate in physiological tissue repair and remodeling. Such precursors then receive signals at extravascular sites of tissue injury (i.e. the heart) that direct their lineage commitment and maturation. In physiologic conditions these cellular responses mediate homeostatic compensation that helps restore normal (pre-pregnancy) cardiac function. However, we submit that in patho-physiological conditions such as in PPCM the disruption of this normal homeostatic process of tissue remodeling contributes to cardiac decompensation and heart failure.

Autoimmune phenomena and pathogenic mechanisms have been observed and proposed for different forms of idiopathic dilated cardiomyopathies (IDCMs) including PPCM (Caforio et al. 1997, Ansari et al.

2002, Sundstrom et al. 2002). Furthermore, autoantibodies to specific cardiac antigens, such as anti-β1-adrenergic receptor and anti-myosin antibodies, have been reported to be directly involved in autoimmune mechanisms of IDCM and PPCM (Warraich et al. 2002, Jahns et al. 2004). A role for DCs in innate immune-mediated mechanisms and in post-infectious autoimmune mechanisms of IDCM has also recently been proposed (Eriksson et al. 2003). Although, the degree to which autoimmune phenomena are involved in PPCM remains unclear, it is of interest that DCs are linked to the interface between innate and adaptive immunity by virtue of their functional maturity.

DCs are highly specialized antigen-presenting cells. Precursor or immature DCs are primed to sense and respond to a wide variety of environmental signals through a broad array of cell surface-expressed chemokine receptors and TLRs. Immature DCs use these receptors to interrogate antigens and to negotiate appropriate immune responses. Immature DCs express germ-line encoded receptors that recognize pathogen-associated molecular patterns (PAMP) expressed on endogenous antigens from damaged cells, e.g. HSPs or certain bacterial or viral antigens. Signaling through such receptors induces DC maturation, including antigen processing, upregulation of major histocompatibility class II molecules, induction of costimulatory activity and migration to regional lymph nodes, where they are able to prime antigen-specific T cells (Srivastava 2002a, b). DC processing and presentation of endogenous antigens released during tissue injury can trigger autoreactive T cells if the DCs are activated inappropriately (Turley 2002). It has been demonstrated in murine models of post-infectious myocarditis that BALB-c mice given DCs preloaded with cardiac specific α-myosin peptides develop cardiomyopathy if DCs are prestimulated with CD40L and TLR agonists. Such experimental DC-mediated myocarditis progressed into dilated cardiomyopathy and heart failure even after resolution of acute inflammatory infiltrates (Eriksson et al. 2003). Examination of explanted hearts (at autopsy or during heart transplant) from PPCM patients in Haiti or in the US as well as endomyocardial biopsies from patients with post-infectious cardiomyopathy do not necessarily show inflammatory infiltrates, even when autoantibodies are present (unpublished observations). Thus, the results from murine studies are consistent with the clinical observations from PPCM subjects and suggest a common pathogenesis of a possible post-infectious form of PPCM.

The hypothesis tested in this investigation was that dysregulation of the expression of factors involved in homeostatic physiological tissue remodeling during the peripartum period would be manifested by a diminished capacity of plasma from subjects with documented PPCM to promote functional maturation of progenitor DCs relative to plasma collected from age and parity matched control subjects. The results from in vitro studies presented in this report convincingly show that a high frequency of PPCM plasma showed a significantly diminished capacity to promote maturation of cord blood derived progenitor DCs relative to plasma obtained from Haitian control subjects as measured by alloproliferative responses by PBMCs (Figure 1B). These results were not due to a loss of viability (data not shown) and the diminished CBDC maturation strongly correlated with the poor expression of MCH class I/II and co-stimulatory molecules, CD80 and 86, as well as DC maturation antigens: CCR7 and CD83 (Figure 2) on cultured prCBDCs. As expected, plasma biomarkers associated with cardiomyopathy and inflammation: endothelin-1, proBNP and CRP, were significantly elevated in PPCM subjects. However, the observation that select cytokines and growth factors involved in DC maturation and innate immune activation were also significantly (GM-CSF; $p < 0.008$) or slightly (TNF-α, $p < 0.08$ and IL-6, $p < 0.06$) elevated in PPCM patients relative to control subjects (Table III) appeared to conflict with data from our functional studies that show evidence of diminished DC maturation (Figures 1A and 2). Surprisingly, cytokines that are known to inhibit immune responses, such as TGF-β, were in fact decreased in the plasma from PPCM patients compared to controls. These data suggest there must be other cytokines and growth factors that override the pro-inflammatory cytokines which need to be identified. It should also be noted that ELISA basically identifies the existence of a protein which may or may not be functional. Thus, it could be that the maturation-inducing cytokines may be dysfunctional. Further studies of the quantitation of biologically functional molecules in PPCM as compared to appropriate normal controls from Haiti need to be performed and are in progress. Furthermore, the fact that DC maturation was observed in *in vitro* culture with plasma from Haitian mothers but not with plasma from matched cord blood or non-Haitian male or nulliparous female volunteers also remains unexplained. However, the question of whether such phenomena are characteristic of all post-partum women or only Haitian mothers was not addressed and was beyond the scope of this investigation. The results of this study describe a unique clinical pattern that reflects an immune environment in which DC maturation and perhaps an even broader potential for critical progenitor cell-mediated tissue repair and remodeling responses are blunted in Haitian PPCM patients. Collectively, these results also support our hypothesis that the absence or suppression of such innate immune-mediated responses contributes to the pathogenesis and irreversible progression towards PPCM in women with early stage heart disease during the peripartum period.

### References

Alonso-Martinez JL, Llorente-Diez B, Echegaray-Agara M, Olaz-Preciado F, Urbieta-Echezarreta M, Gonzalez-Arencibia C. 2002. C-reactive protein as a predictor of improvement and readmission in heart failure. Eur J Heart Fail 4:331–336.

Altieri P, Brunelli C, Garibaldi S, Nicolino A, Ubaldi S, Spallarossa P, Olivotti L, Rossettin P, Barsotti A, Ghigliotti G. 2003. Metalloproteinases 2 and 9 are increased in plasma of patients with heart failure. Eur J Clin Investig 33:648–656.

Anker SD, von Haehling S. 2004. Inflammatory mediators in chronic heart failure: An overview. Heart 90:464–470.

Ansari AA, Fett JD, Carraway RE, Mayne AE, Onlamoon N, Sundstrom JB. 2002. Autoimmune mechanisms as the basis for human peripartum cardiomyopathy. Clin Rev Allergy Immunol 23:301–324.

Aukrust P, Ueland T, Lien E, Bendtzen K, Muller F, Andreassen AK, Nordoy I, Aass H, Espevik T, Simonsen S, Froland SS, Gullestad L. 1999. Cytokine network in congestive heart failure secondary to ischemic or idiopathic dilated cardiomyopathy. Am J Cardiol 83:376–382.

Brown CS, Bertolet BD. 1998. Peripartum cardiomyopathy: A comprehensive review. Am J Obstet Gynecol 178:409–414.

Bukau B, Horwich AL. 1998. The Hsp70 and Hsp60 chaperone machines. Cell 92:351–366.

Caforio AL, Bauce B, Boffa GM, De Cian F, Angelini A, Melacini P, Razzolini R, Fasoli G, Chioin R, Schiaffino S, Thiene G, Dalla VS. 1997. Autoimmunity in myocarditis and dilated cardiomyopathy: Cardiac autoantibody frequency and clinical correlates in a patient series from Italy. G Ital Cardiol 27:106–112.

Eriksson U, Ricci R, Hunziker L, Kurrer MO, Oudit GY, Watts TH, Sonderegger I, Bachmaier K, Kopf M, Penninger JM. 2003. Dendritic cell-induced autoimmune heart failure requires cooperation between adaptive and innate immunity. Nat Med 9:1484–1490.

Fett JD, Carraway RD, Dowell DL, King ME, Pierre R. 2002. Peripartum cardiomyopathy in the Hospital Albert Schweitzer District of Haiti. Am J Obstet Gynecol 186:1005–1010.

Flesch M, Hoper A, Dell'Italia L, Evans K, Bond R, Peshock R, Diwan A, Brinsa TA, Wei CC, Sivasubramanian N, Spinale FG, Mann DL. 2003. Activation and functional significance of the renin-angiotensin system in mice with cardiac restricted overexpression of tumor necrosis factor. Circulation 108:598–604.

Hendrick JP, Hartl FU. 1995. The role of molecular chaperones in protein folding. FASEB J 9:1559–1569.

Inoue T, Kato T, Takayanagi K, Uchida T, Yaguchi I, Kamishirado H, Morooka S, Yoshimoto N. 2003. Circulating matrix metalloproteinase-1 and -3 in patients with an acute coronary syndrome. Am J Cardiol 92:1461–1464.

Jahns R, Boivin V, Hein L, Triebel S, Angermann CE, Ertl G, Lohse MJ. 2004. Direct evidence for a beta 1-adrenergic receptor-directed autoimmune attack as a cause of idiopathic dilated cardiomyopathy. J Clin Investig 113:1419–1429.

Kai H, Ikeda H, Yasukawa H, Kai M, Seki Y, Kuwahara F, Ueno T, Sugi K, Imaizumi T. 1998. Peripheral blood levels of matrix metalloproteases-2 and -9 are elevated in patients with acute coronary syndromes. J Am Coll Cardiol 32:368–372.

Kaneko K, Kanda T, Yamauchi Y, Hasegawa A, Iwasaki T, Arai M, Suzuki T, Kobayashi I, Nagai R. 1999. C-Reactive protein in dilated cardiomyopathy. Cardiology 91:215–219.

Kaneko K, Kanda T, Hasegawa A, Suzuki T, Kobayashi I, Nagai R. 2000. C-reactive protein as a prognostic marker in lymphocytic myocarditis. Jpn Heart J 41:41–47.

Knowlton AA, Kapadia S, Torre-Amione G, Durand JB, Bies R, Young J, Mann DL. 1998. Differential expression of heat shock proteins in normal and failing human hearts. J Mol Cell Cardiol 30:811–818.

Kuppner MC, Gastpar R, Gelwer S, Nossner E, Ochmann O, Scharner A, Issels RD. 2001. The role of heat shock protein (hsp70) in dendritic cell maturation: hsp70 induces the maturation of immature dendritic cells but reduces DC differentiation from monocyte precursors. Eur J Immunol 31:1602–1609.

Latif N, Baker CS, Dunn MJ, Rose ML, Brady P, Yacoub MH. 1993. Frequency and specificity of antiheart antibodies in patients with dilated cardiomyopathy detected using SDS-PAGE and western blotting. J Am Coll Cardiol 22:1378–1384.

Latif N, Taylor PM, Khan MA, Yacoub MH, Dunn MJ. 1999. The expression of heat shock protein 60 in patients with dilated cardiomyopathy. Basic Res Cardiol 94:112–119.

Mandi Y, Hogye M, Talha EM, Skolak E, Csanady M. 2000. Cytokine production and antibodies against heat shock protein 60 in cardiomyopathies of different origins. Pathobiology 68:150–158.

Mann DL. 1996. Stress activated cytokines and the heart. Cytokine Growth Factor Rev 7:341–354.

Mann DL. 2002a. Angiotensin II as an inflammatory mediator: Evolving concepts in the role of the renin angiotensin system in the failing heart. Cardiovasc Drugs Ther 16:7–9.

Mann DL. 2002b. Inflammatory mediators and the failing heart: Past, present, and the foreseeable future. Circ Res 91:988–998.

Mann DL. 2003. Stress-activated cytokines and the heart: From adaptation to maladaptation. Annu Rev Physiol 65:81–101.

Portig I, Pankuweit S, Maisch B. 1997. Antibodies against stress proteins in sera of patients with dilated cardiomyopathy. J Mol Cell Cardiol 29:2245–2251.

Schwartzkopff B, Fassbach M, Pelzer B, Brehm M, Strauer BE. 2002. Elevated serum markers of collagen degradation in patients with mild to moderate dilated cardiomyopathy. Eur J Heart Fail 4:439–444.

Srivastava P. 2002a. Interaction of heat shock proteins with peptides and antigen presenting cells: chaperoning of the innate and adaptive immune responses. Annu Rev Immunol 20:395–425.

Srivastava P. 2002b. Interaction of heat shock proteins with peptides and antigen presenting cells: chaperoning of the innate and adaptive immune responses. Annu Rev Immunol 20:395–425.

Stumpf C, Lehner C, Eskafi S, Raaz D, Yilmaz A, Ropers S, Schmeisser A, Ludwig J, Daniel WG, Garlichs CD. 2003. Enhanced levels of CD154 (CD40 ligand) on platelets in patients with chronic heart failure. Eur J Heart Fail 5:629–637.

Sundstrom JB, Fett JD, Carraway RD, Ansari AA. 2002. Is peripartum cardiomyopathy an organ-specific autoimmune disease? Autoimmun Rev 1:73–77.

Suzuki T, Yamazaki T, Yazaki Y. 2001. The role of the natriuretic peptides in the cardiovascular system. Cardiovasc Res 51:489–494.

Turley SJ. 2002. Dendritic cells: inciting and inhibiting autoimmunity. Curr Opin Immunol 14:765–770.

Ventura SJ, Peters KD, Martin JA, Maurer JD. 1997. Births and deaths: United States, 1996. Mon Vital Stat Rep 46:1–40.

Warraich RS, Noutsias M, Kazak I, Seeberg B, Dunn MJ, Schultheiss HP, Yacoub MH, Kuhl U, Kasac I. 2002. Immunoglobulin G3 cardiac myosin autoantibodies correlate with left ventricular dysfunction in patients with dilated cardiomyopathy: Immunoglobulin G3 and clinical correlates. Am Heart J 143:1076–1084.

Yamazaki T, Lee JD, Shimizu H, Uzui H, Ueda T. 2004. Circulating matrix metalloproteinase-2 is elevated in patients with congestive heart failure. Eur J Heart Fail 6:41–45.

Zolk O, Quattek J, Seeland U, El-Armouche A, Eschenhagen T, Bohm M. 2002. Activation of the cardiac endothelin system in left ventricular hypertrophy before onset of heart failure in TG(mREN2)27 rats. Cardiovasc Res 53:363–371.

